# Optimizing Thyroxine Dosage After Total Thyroidectomy: Understanding the Factors at Play

**DOI:** 10.7759/cureus.58430

**Published:** 2024-04-16

**Authors:** Edwin Dawn Paul, Santhosh T V, Sumin V Sulaiman

**Affiliations:** 1 General Surgery, Government Medical College Thrissur, Thrissur, IND

**Keywords:** thyroxine dose, total thyroidectomy, benign thyroid disorders, lbm, bsa, bmi, t4 dose

## Abstract

Introduction: Total thyroidectomy is evolving as the choice of treatment for non-malignant thyroid conditions. Therefore, an ideal method of thyroxine replacement is necessary to avoid the ill effects of under- and over-replacement in such patients.

Aim: To assess the correlation between optimal thyroxine dose and potential variables like lean body mass (LBM), body surface area (BSA), body mass index (BMI), body weight, age, and sex in patients who underwent total thyroidectomies for benign multinodular goiters in our institute.

Materials and methods: A longitudinal cohort study was undertaken at the Government Medical College Thrissur, a tertiary care provider in India, between October 2018 and September 2019. One hundred adult patients who underwent a total thyroidectomy for various benign thyroid conditions were included. They were initially given thyroxine 75 µg upon discharge and received follow-up doses every two months until they achieved euthyroid status on two consecutive visits. The variables evaluated at this stage included age, sex, actual body weight, lean body weight, BMI, and biochemical data (triiodothyronine (T3), thyroxine (T4), thyroid-stimulating hormone (TSH)). Correlation, multiple step-wise regression, and variance were carried out using EPI INFO version 7.2.2.6.

Results: The best predictors for optimum thyroxine dose were BSA (0.923, P < 0.01) and LBM (0.921, P < 0.01), compared with body weight (0.833, P < 0.01) and BMI (0.523, P < 0.01). In our study, the least significant factor was the age of the patient (r = 0.117, P < 0.01). There was no significant association between gender and thyroxine dose. The mean thyroxine dose was 1.87 µg/kg of the patient’s body weight.

Conclusion: The optimum thyroxine replacement based on BSA or LBM is a more ideal method than based on BMI or body weight alone.

## Introduction

Recently, total thyroidectomy has been recommended as the optimal surgical treatment for benign bilateral multinodular goiters [1]. In these patients, thyroxine (T4) replacement is the standard of care after surgery. It is essential to achieve a clinical and biochemical euthyroid state as early as possible to prevent the occurrence of hypothyroidism or hyperthyroidism. For this reason, a precise calculation of the thyroxine dose is critical.

Traditionally, thyroxine replacement is based on body weight (per kg/day), and the usual dose is 1.6 µg/kg/day, which is mainly intended for patients with primary hypothyroidism [2]. This dose might not be right for people who have had a thyroidectomy for two reasons: first, their bodies do not make thyroxine like they do in primary hypothyroidism; and second, the literature on the T4 requirement based on body weight ranges widely (from 1.5 to 2.04 mcg/kg/day) [3,4].

Several factors other than body weight influence the optimal thyroxine requirement. Lean body mass (LBM) and body surface area (BSA) are suggested as more accurate predictors than actual body weight in the literature [4]. Age and sex also influence the final dose of thyroxine required [5,6]. Unfortunately, there is no consensus as to which is the best predictor.

Data regarding optimal thyroxine requirements in the Indian population are also very limited [4]. The objective of this study is to evaluate the correlation between optimal thyroxine replacement dose and multiple parameters like body weight, BSA, LBM, age, and sex in individuals who have undergone total thyroidectomies for benign multi-nodular goiters in our institute.

## Materials and methods

This was a hospital-based longitudinal cohort study conducted in the Department of General Surgery, Government Medical College Thrissur, Thrissur, India. The Institutional Ethical Committee granted its approval with order no. B6/8772/2016/MCTCR (20) on November 22, 2017. The study period ranged from October 1, 2018, to September 30, 2019.

Inclusion and exclusion criteria

Patients who underwent total thyroidectomies for various benign thyroid conditions in the 15-70 age group, both sexes, from October 1, 2018, to September 30, 2019, were included in the study. Patients with malignancy, acute infections, adrenal disorders, pituitary abnormalities, and those taking medications like iron, calcium, antacids, and PPIs were excluded.

The sample size was 100, based on an article by Mistry et al. [5].

All the patients were given thyroxine 75 µg on discharge and followed up every two months as per department policy until euthyroid status was achieved. During the follow-up period, patients underwent clinical examinations and a thyroid function test to assess their progress. Based on the results, appropriate adjustments were made to their medication dosage. A patient showing stable biochemical euthyroid status on two consecutive visits was included in our study. Euthyroid status defined for the purpose of the study was a thyroid-stimulating hormone (TSH) level between 0.4 and 4 IU/ml and a normal free T4 level [7]. We assessed 164 patients, and of these, 100 met our criteria. The variables recorded at this stage included age, sex, actual body weight, lean body weight, body mass index (BMI), and biochemical data (triiodothyronine (T3), thyroxine (T4), and TSH). In our study, LBM was calculated using the Hume formula [8] (Male LBM = (0.328 x weight in kg) + (0.339 x height in cm) - 29.533; Female LBM = (0.295 x weight in kg) + (0.418 x height in cm) - 43.293). BSA was calculated using the DuBois formula [9] (BSA(m^2^) = 0.202 x height (m) 0.725 x weight (kg) 0.425).

Statistical analysis

The data were coded and entered in Microsoft Excel. The mean, standard deviation, median, and interquartile ranges were used to express quantitative variables. The association between quantitative variables was analyzed using correlation regression and multiple regressions. Data were presented as mean ± SD. P values <0.05 were considered statistically significant. The statistical program EPI INFO version 7.2.2.6 (Centers for Disease Control and Prevention, Atlanta, United States), was used for the analysis.

## Results

A total of 100 patients who underwent total thyroidectomies for benign thyroid conditions in our tertiary center were evaluated and analyzed for this study after obtaining informed written consent. There were 13 men and 87 women (M:F = 1:6.7). The mean age of the 100 patients in the study group was 42.27 ± 11.67 (range: 20-69; Table 1).

**Table 1:** Age distribution

Age distribution	No. of patients
<30	14
31-40	35
41-50	30
51-60	13
>61	8

Among 100 cases, 41 patients had nodular colloid goiters, 33 patients had Hashimoto’s thyroiditis, 17 patients had lymphocytic thyroiditis, and the rest (nine patients) had follicular adenoma (Table 2).

**Table 2:** Case distribution

Histological variants	Case distribution
Nodular colloid goiter	41
Hashimoto’s thyroiditis	33
Lymphocytic thyroiditis	17
Follicular adenoma	9
Total	100

The mean thyroxine dose per kilogram (kg) of body weight was 1.87 µg/kg (standard error of mean 0.61). The mean thyroxine dose per LBM was 2.79 µg/kg (standard error of mean 0.9). The mean thyroxine dose per BSA was 72.87 µg/m^2^ (standard error of mean 21.5). The mean thyroxine dose per BMI was 4.96 µg/kg (standard error of mean 1.63).

The mean body weight of patients in this study group was 66.42 ± 12.61 (range: 45-98), mean BMI was 25.48 ± 4.27 (range: 17.36-35.42), mean BSA was 1.698 ± 0.172 (range: 1.434-2.246), and mean LBM was 44.15 ± 6.44 (range: 33.67-66.41) (Table 3).

**Table 3:** Multivariate analysis using multiple correlation-regression BMI: Body mass index; BSA: Body surface area; LBM: Lean body mass

Parameters	Mean ± SD	Correlation	P-value
Age	42.45 ± 11.67	0.117	0.248
Weight	66.42 ± 12.61	0.833	<0.01
BMI	25.48 ± 4.27	0.523	<0.01
BSA	1.698 ± 0.172	0.923	<0.01
LBM	44.15 ± 6.44	0.921	<0.01

The thyroxine replacement dose was correlated with multiple factors. BMI (0.523, P < 0.01), body weight (0.833, P < 0.01), LBM (0.921, P < 0.01), and BSA (0.923, P < 0.01) were found to be significantly correlated with the final thyroxine dose. Statistically, LBM and BSA were found to be more closely related to the final thyroxine dose (Table 3). There was no significant association between age and the final thyroxine dose.

**Figure 1:**
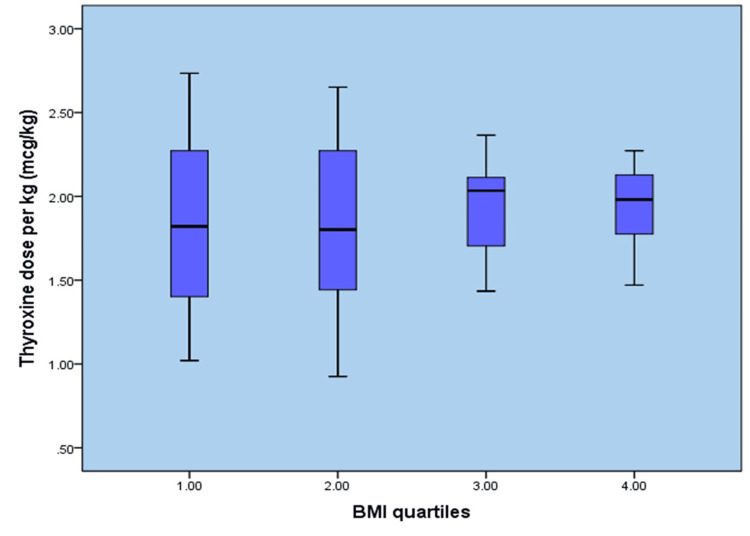
Body mass index versus thyroxine dose (µg/kg) BMI: Body mass index

**Figure 2:**
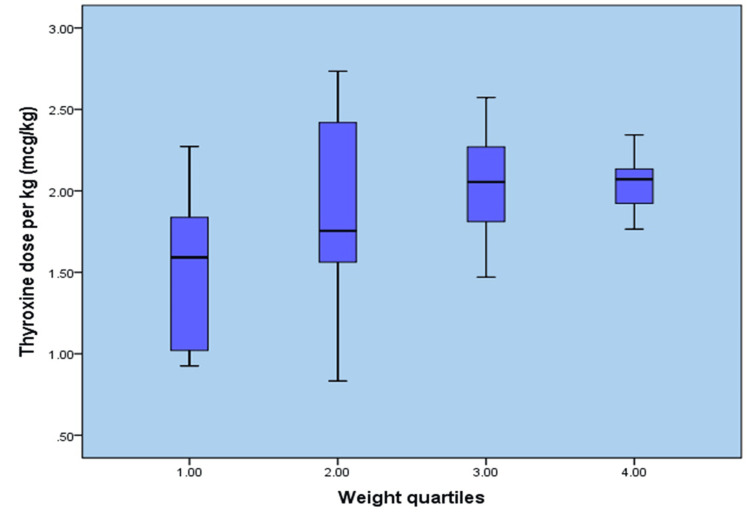
Body weight in kg versus final thyroxine dose (µg/kg)

**Figure 3:**
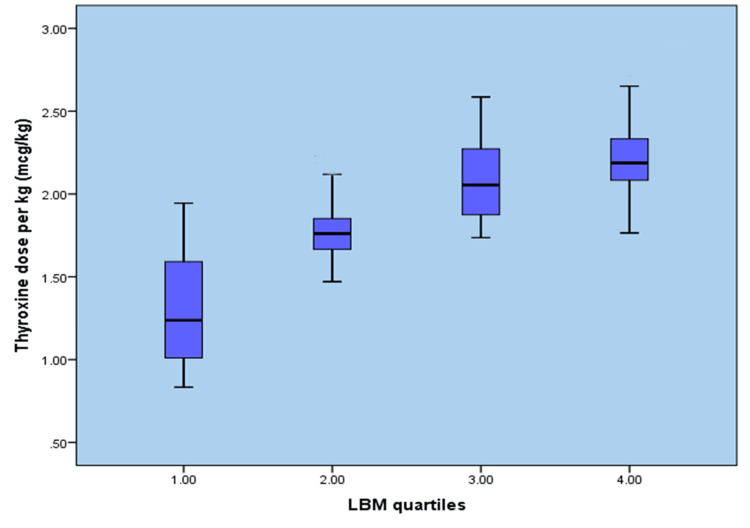
Lean body mass versus final thyroxine dose (µg/kg) LBM: Lean body mass

**Figure 4:**
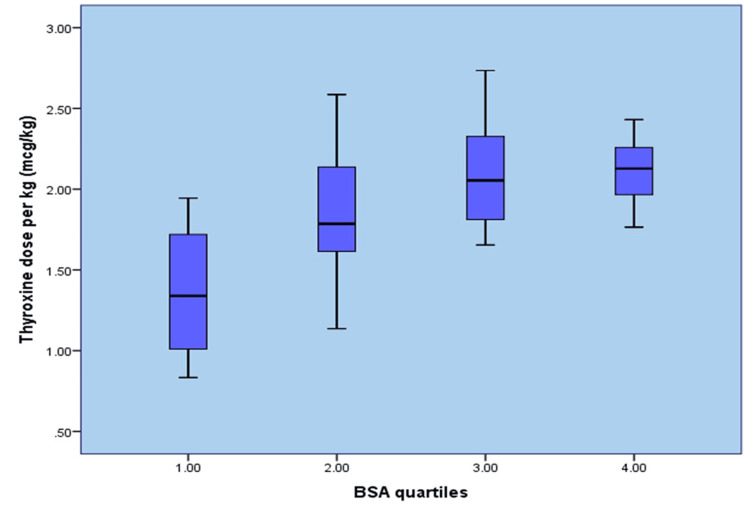
Body surface area versus final thyroxine dose (µg/kg) BSA: Body surface area

There was an incidental increase in thyroxine dose along with increases in body weight, BSA, and LBM, but not with an increase in BMI (Figures 1-4). When the entire group of patients was examined for a correlation between BMI and thyroxine dose, our study failed to demonstrate a linear increase in thyroxine dosage corresponding to a rise in BMI values, as shown in Figure 1.

Similarly, the entire division was examined for a correlation between the required final thyroxine dose and the patient’s body weight. As shown in Figure 2, the thyroxine requirement increased with weight, indicating a moderate correlation between the two variables.

In contrast to other variables, the quartiles of LBM and BSA showed a significant correlation with the final thyroxine dose by demonstrating a linear increase in thyroxine requirement with an increase in LBM and BSA, as shown in Figures 3, 4.

**Figure 5:**
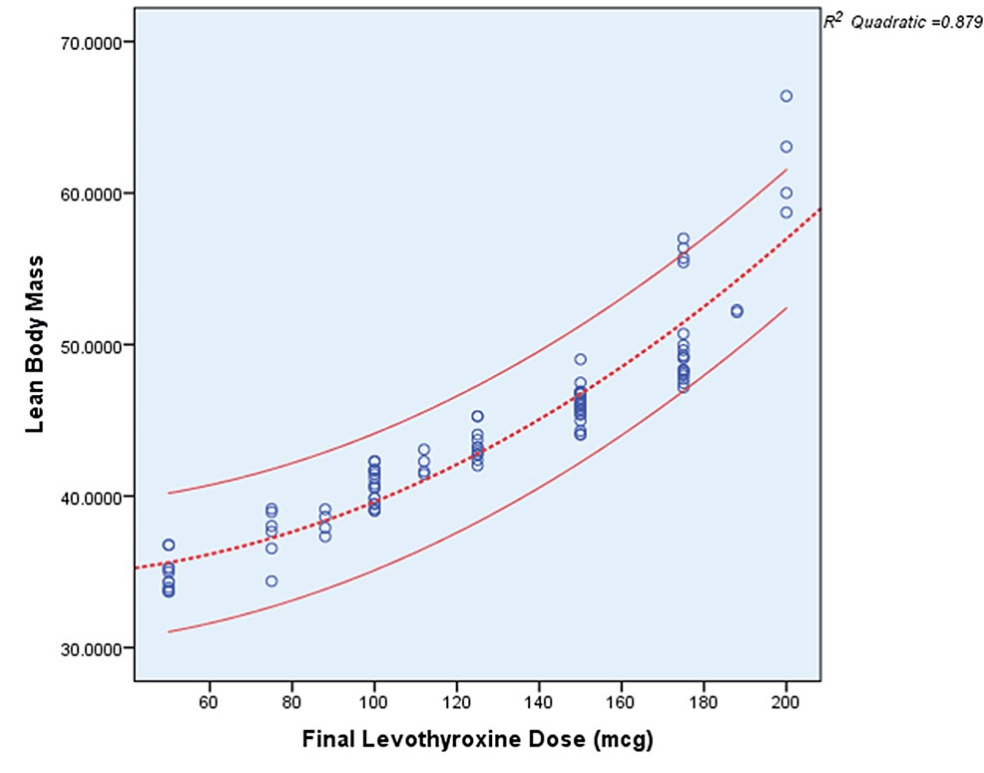
Scatter plot between final T4 dose and lean body mass T4: Thyroxine

**Figure 6:**
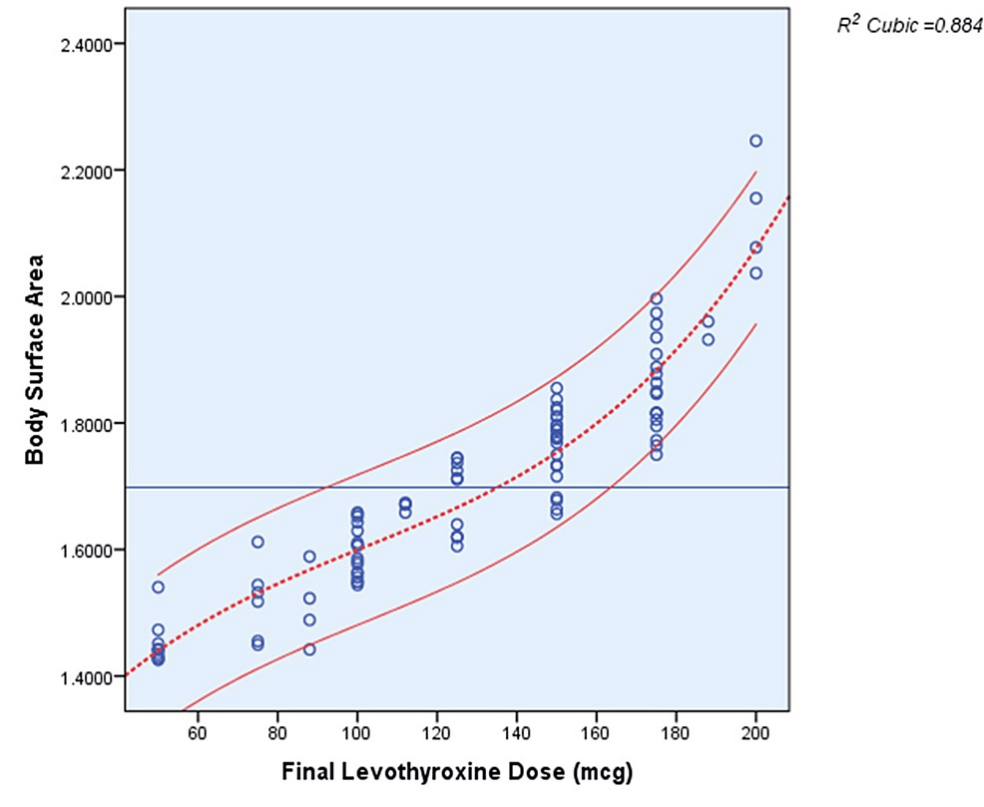
Scatter plot between final T4 dose and body surface area T4: Thyroxine

Among the multiple analyses and correlations performed in this cohort, the best R^2^ scores of 0.879 and 0.884 were obtained in relation to LBM and BSA, which gave a better prediction for the final thyroxine dose requirement after a total thyroidectomy for benign thyroid disorders (Figures 5, 6).

## Discussion

We found that LBM and BSA strongly correlate with thyroxine requirements. These two factors also exhibit linear relationships with thyroxine requirements, i.e., an incremental increase in thyroxine requirements with an increase in LBM and BSA. Actual body weight also correlates with thyroxine requirements, but the correlation is weak.

The usual practice is to replace thyroxine after a thyroidectomy with a dose of 1.6 µg/kg [2,7]. This dosage schedule was borrowed from the experience of treating primary hypothyroidism. In primary hypothyroidism, there may be some residual endogenous production of thyroxine. So, this dose may not be adequate for post-total thyroidectomy patients.

In the present study, the thyroxine dose based on body weight was 1.87 (±0.61) µg/kg, which is higher than the usual recommended dosage of 1.6 µg/kg. This was also higher than the usual reported dosage from the West (1.5-1.6 µg/kg) in total thyroidectomy patients [3].

Calculating the thyroxine dose based on body weight has some limitations. Because adipose tissues are metabolically less active, the thyroxine requirement of fat cells is minimal compared to highly metabolically active tissues like the muscles, brain, and kidney. So, it is assumed that the total thyroxine requirement is directly proportional to the LBM rather than the whole-body weight.

BMI

According to a retrospective study by Jin et al. [3], patients with a BMI under 30 should take 1.5 µg/kg of thyroxine, whereas those with a BMI over 30 should take 1.4 µg/kg. In a retrospective investigation of 98 patients who underwent a total thyroidectomy, Olubowale et al. [10] discovered that the final thyroxine dose was significantly influenced by age, body weight, and BMI. There was a significant correlation (r = 0.46, P < 0.001) between body weight and T4 dosage. They have also seen a consistent relationship between body weight and the proper T4 needs. The median thyroxine dose was 1.69 µg/kg; however, a greater dose (2.12 µg/kg/day) was needed in individuals who were slimmer, meaning they weighed less than 60 kg. The mean BMI in both investigations (32 kg/m^2^ and 27 kg/m^2^ vs. 25 kg/m^2^) was greater than the mean BMI of the study population.

Our study analysis agrees with Jonklaas’s, in which the daily thyroxine requirement was 1.8 µg/kg with a mean BMI of 27 kg/m^2^ [7]. Meanwhile, a study from the UK revealed a thyroxine dose of 2 µg/kg with a similar body weight profile as our community, with a mean BMI of 27 kg/m^2^ [5]. However, a randomized study from India had a similar thyroxine dose of 2.04 µg/kg with a mean BMI of 22.67 kg/m^2^, showing an incremental increase in thyroxine dose with an increase in body weight, as in our analysis [4].

In a retrospective study, Ojomo et al. [11] concluded that BMI was a more accurate determinant than body weight and suggested a dosage model based on BMI. According to them, body weight-based thyroxine dosage achieved euthyroid status in only 39% of patients on their first visit, which led to overdose in patients with BMIs < 25 kg/m^2^ (46%) and underdose in patients with BMIs > 30 kg/m^2^ (50%).

A recent retrospective data analysis from obese and overweight thyroidectomy patients also reported similar observations. A replacement dose based on body weight led to overdosage on their first visit compared to BMI-based dosing. Both the BMI and body weight-based calculations needed an incremental scaling of thyroxine dose to achieve euthyroid status; the increase was greater in the body weight group, causing hyperthyroidism [12].

An explanation for the higher thyroxine requirement in the Indian population may be the high prevalence of *Helicobacter pylori*-related chronic gastritis [13,14]. The prevalence of *H. pylori* infections in our population was 80-90%. *H. pylori* tends to decrease the oral absorption of thyroxine by decreasing gastric acid secretion in the stomach. In a prospective, non-randomized trial, *H. pylori* eradication led to a lowering of TSH levels from 30.5 international units (IU) to 4.2 IU in patients who were non-responsive to thyroxine treatment. Similarly, the requirement for thyroxine was high in individuals with* H. pylori*-associated gastritis compared to individuals without *H. pylori*-related gastritis (2.05 µg/kg/day vs. 1.5 µg/kg/day) [13].

LBM

It is logical to assume that LBM is a better predictor than actual weight because muscles are more metabolically active than adipose tissues and bones. There are studies on primary hypothyroidism that concur with these assumptions [15]. However, some studies failed to prove the correlation between LBM and thyroxine dose in total thyroidectomy patients. For example, Olubowale et al. [10] failed to demonstrate a significant linear correlation between T4 requirements and LBM estimated using anthropometry. Sukumar et al. [4] used the X-ray impedance method to estimate LBM in their study, but when they used multivariate analysis, they failed to demonstrate a correlation between LBM and T4 replacement dose in a total thyroidectomy.

Santini et al. [16] reported a strong correlation between the suppressive dose of thyroxine and LBM (P = 0.0001, r = 0.66) in patients following a total thyroidectomy for a well-differentiated thyroid malignancy. They assessed body mass using DEXA scans in 75 patients, and their endpoint was a suppressive dose of T4 to achieve a TSH level between 0.005 µIU/L and 0.3 µIU/L and normal FT_4_ and FT_3_.

In the interim, our research demonstrated a noteworthy linear association between LBM and thyroxine replacement dose among participants who underwent total thyroidectomy.

The mean thyroxine requirement of the present study was 2.79 µg/kg of LBM, which is higher than the conventionally suggested dose of 2.5 µg/kg of LBM. In primary hyperthyroidism, a thyroxine replacement dose of 2.3 µg/kg of LBM was suggested [17]. One of the limitations of LBM is that its measurement is cumbersome in clinical practice. Either we must use complicated anthropometric formulae or specialized machines like dual-absorption X-ray densitometers, so using body weight is a simpler and more useful routine practice. In the present study, we estimated the LBM based only on routine anthropometric measurements and the aforementioned formula. Compared with other factors, LBM visualized a good R^2^ value of 0.921 during our analysis of the thyroxine dose.

BSA

Another interesting finding of our study was a strong correlation between thyroxine requirements and BSA. Among multiple factors, BSA showed the best R^2^ value of 0.923 in correlation with the final thyroxine dose. Here, the calculated mean thyroxine dose requirement based on BSA was 72.3 µg/m^2^/day. Another randomized trial performed in 2010 also came up with a similar interpretation in which the mean thyroxine requirement was 75.2 µg/m^2^/day of BSA [4].

A retrospective analysis of data from 234 post-thyroidectomy patients stated that BSA was the most important predictor of optimum T4 thyroxine. A patient with a BSA of more than 1.79 m^2 ^needed a dose of 1.4 µg/kg/day, whereas patients with a BSA of less than 1.79 m^2^ required a dose of 1.7 µg/kg/day [18].

Age-related decreases in thyroxine replacement dosages for primary hypothyroidism have been shown in Western studies [5]. Nevertheless, our research revealed a negligible relationship between age and thyroxine dosage. Because of the limited number of men in our analysis, there was not enough data to draw solid conclusions about the impact of sex on thyroxine replacement therapy.

It was discovered that replacing thyroxine based on LBM or BSA was better than replacing it just on body weight or BMI.

Limitations

One of the study's weaknesses is the limited amount of data on older people due to the tiny proportion of the elderly community. Due to the limited percentage of men (1:6.7) in the study sample, we were unable to reach a firm conclusion about the impact of gender on levothyroxine replacement treatment. Even though we were only able to employ anthropometric measurements of LBM due to the unavailability of objective measurements, we were nevertheless able to find a strong association between LBM and levothyroxine dose.

## Conclusions

Calculations based on BSA or LBM give a more accurate dosage schedule for T4 than those based on body weight in post-thyroidectomy patients. Our study may provide preliminary insight into the calculation of thyroxine replacement therapy. Caregivers must focus on achieving euthyroid status as early as possible to improve the patient’s compliance by reducing the need for outpatient attendance for dose titration and escalating the cost-effectiveness of treatment. Therefore, further randomized control trials are necessary to confirm our findings in this study.
